# Supplementary material for: Impact of Periodontal Attachment Loss on the Outcome of Endodontic Microsurgery: A Systematic Review and Meta-Analysis
Source: Medicina (Kaunas). 2021 Sep 1;57(9):922. doi: 10.3390/medicina57090922 (PMC8465214; doi:10.3390/medicina57090922)
Supplement: Supplementary file 1 [file medicina-57-00922-s001.zip › Table S1.pdf]

Table S1. Kim and Kratchman's classification of lesion types in endodontic surgical cases [12].

Class	Description
A	Absence of a periapical lesion, no mobility and normal probing depth, unresolved symptoms after nonsurgical therapies.
B	The presence of a small periapical lesion as well as clinical symptoms. Tooth with normal periodontal probing depth and no mobility.
C	Large periapical lesion progressing coronally without periodontal pocket and mobility.
D	Clinically similar to class C but with deep periodontal pockets.
E	Deep periapical lesion with an endodontic-periodontal communication to the apex and no obvious fracture.
F	Tooth with an apical lesion, complete absence of the buccal plate and no mobility.
