# Supplementary material for: Impact of Periodontal Attachment Loss on the Outcome of Endodontic Microsurgery: A Systematic Review and Meta-Analysis
Source: Medicina (Kaunas). 2021 Sep 1;57(9):922. doi: 10.3390/medicina57090922 (PMC8465214; doi:10.3390/medicina57090922)
Supplement: Supplementary file 1 [file medicina-57-00922-s001.zip › Table S2.pdf]

Table S2. Excluded studies after full-text reading.

	Study	Exclusion criteria
**1**	Azim AA, Albanyan H, Azim KA, Piasecki L. The Buffalo study: outcome and associated predictors in endodontic microsurgery- a cohort study. International Endodontic Journal. 2021 Mar;54(3):301–18.	2
**2**	Wang ZH, Zhang MM, Wang J, Jiang L, Liang YH. Outcomes of endodontic microsurgery using a microscope and mineral trioxide aggregate: a prospective cohort study. Journal of Endodontics. 2017 May;43(5):694-698.	10
**3**	Kim S, Song M, Shin SJ, Kim E. A randomized controlled study of mineral trioxide aggregate and super ethoxybenzoic acid as root-end filling materials in endodontic microsurgery: long-term outcomes. Journal of Endodontics. 2016 Jul;42(7):997-1002.	2
**4**	Kim D, Ku H, Nam T, Yoon TC, Lee CY, Kim E. Influence of size and volume of periapical lesions on the outcome of endodontic microsurgery: 3-dimensional analysis using cone-beam computed tomography. Journal of Endodontics. 2016 Aug;42(8):1196–201.	10
**5**	Song M, Nam T, Shin SJ, Kim E. Comparison of clinical outcomes of endodontic microsurgery: 1 year versus long-term follow-up. Journal of Endodontics. 2014 Apr;40(4):490–4.	10
**6**	Shinbori N, Grama AM, Patel Y, Woodmansey K, He J. Clinical outcome of endodontic microsurgery that uses endosequence bc root repair material as the root-end filling material. Journal of Endodontics. 2015 May;41(5):607–12.	10
**7**	Song M, Kim E. A prospective randomized controlled study of mineral trioxide aggregate and super ethoxy-benzoic acid as root-end filling materials in endodontic microsurgery. Journal of Endodontics. 2012 Jul;38(7):875–9.	2
**8**	Albanyan H, Aksel H, Azim AA. Soft and hard tissue remodeling after endodontic microsurgery: a cohort study. Journal of Endodontics. 2020 Dec;46(12):1824–31.	2,8
**9**	Taha NA, Aboyounes FB, Tamimi ZZ. Root-end microsurgery using a premixed tricalcium silicate putty as root-end filling material: a prospective study. Clinical Oral Investigations. 2021 Jan;311–317.	1,10
**10**	Chan S, Glickman GN, Woodmansey KF, He J. Retrospective analysis of root-end microsurgery outcomes in a postgraduate program in endodontics using calcium silicate–based cements as root-end filling materials. Journal of Endodontics. 2020 Mar;46(3):345–51.	2
**11**	Karan NB, Aricioğlu B. Assessment of bone healing after mineral trioxide aggregate and platelet-rich fibrin application in periapical lesions using cone-beam computed tomographic imaging. Clinical Oral Investigations. 2020 Feb;24(2):1065–72.	2,6
**12**	Tawil PZ, Saraiya VM, Galicia JC, Duggan DJ. Periapical microsurgery: the effect of root dentinal defects on short- and long-term outcome. Journal of Endodontics. 2015 Jan;41(1):22–7.	2
**13**	Taschieri S, del Fabbro M. Endoscopic endodontic microsurgery: 2-year evaluation of healing and functionality. Brazilian Oral Research. 2009 Jan-Mar;23(1):23–30.	10

Table S2. (Continued) Excluded studies after full-text reading.

	Study	Exclusion criteria
**14**	Song M, Shin SJ, Kim E. Outcomes of endodontic micro-resurgery: a prospective clinical study. Journal of Endodontics. 2011 Mar;37(3):316–20.	2
**15**	Schloss T, Sonntag D, Kohli MR, Setzer FC. A comparison of 2- and 3-dimensional healing assessment after endodontic surgery using cone-beam computed tomographic volumes or periapical radiographs. Journal of Endodontics. 2017 Jul;43(7):1072–9.	2
**16**	Li H, Zhai F, Zhang R, Hou B. Evaluation of microsurgery with supereba as root-end filling material for treating post-treatment endodontic disease: a 2-year retrospective study. Journal of Endodontics. 2014 Mar;40(3):345–50.	2
**17**	Pallarés-Serrano A, Glera-Suarez P, Tarazona-Alvarez B, Peñarrocha-Diago M, Peñarrocha-Diago M, Peñarrocha-Oltra D. Prognostic factors after endodontic microsurgery: a retrospective study of 111 cases with 5 to 9 years of follow-up. Journal of Endodontics. 2021 Mar;47(3):397–403.	2
**18**	Kang S, Ha SW, Kim U, Kim S, Kim E. A one-year radiographic healing assessment after endodontic microsurgery using cone-beam computed tomographic scans. Journal of Clinical Medicine. 2020 Nov;9(11):3714.	2
**19**	Safi C, Kohli MR, Kratchman SI, Setzer FC, Karabucak B. Outcome of endodontic microsurgery using mineral trioxide aggregate or root repair material as root-end filling material: a randomized controlled trial with cone-beam computed tomographic evaluation. Journal of Endodontics. 2019 Jul;45(7):831–9.	2
**20**	Shen J, Zhang H, Gao J, Du X, Chen Y, Han L. Short-term observation of clinical and radiographic results of periapical microsurgery: a prospective study. Biomedical Research. 2016 Mar;27(3):923–8.	10
**21**	Kim D, Kim S, Song M, Kang DR, Kohli MR, Kim E. Outcome of endodontic micro-resurgery: a retrospective study based on propensity score–matched survival analysis. Journal of Endodontics. 2018 Nov;44(11):1632–40.	8
