# Supplementary material for: Impact of Periodontal Attachment Loss on the Outcome of Endodontic Microsurgery: A Systematic Review and Meta-Analysis
Source: Medicina (Kaunas). 2021 Sep 1;57(9):922. doi: 10.3390/medicina57090922 (PMC8465214; doi:10.3390/medicina57090922)
Supplement: Supplementary file 1 [file medicina-57-00922-s001.zip › Table S3.pdf]

Table S3. Risk-of-bias of randomized controlled trials based on RoB 2 tool, as recommended by Cochrane.

Study	D1	D2	D3	D4	D5	Overall risk of bias
	Randomization process	Deviations from the intended interventions	Missing outcome data	Measurement of the outcome	Selection of the reported result	
Zhou et al. 2017 [8]	LOW	LOW	LOW	LOW	LOW	LOW
