# Supplementary material for: Impact of Periodontal Attachment Loss on the Outcome of Endodontic Microsurgery: A Systematic Review and Meta-Analysis
Source: Medicina (Kaunas). 2021 Sep 1;57(9):922. doi: 10.3390/medicina57090922 (PMC8465214; doi:10.3390/medicina57090922)
Supplement: Supplementary file 1 [file medicina-57-00922-s001.zip › Table S4.pdf]

Table S4. Risk of bias of cohort studies based on the Newcastle-Ottawa Scale.

Study	Selection	Comparability	Outcome	Overall merit
von Arx et al. 2007 [14]	★★★★	★	★★★	8 stars
Kim E et al. 2008 [15]	★★★★	★	★★★	8 stars
Song et al. 2013 [51]	★★★★	★	★★★	8 stars
Song et al. 2013 [13]	★★★★	★	★★★	8 stars
Song et al. 2011 [26]	★★★★	★	★★★	8 stars
von Arx et al. 2012 [53]	★★★★	★	★★★	8 stars
Song et al. 2012 [9]	★★★★	★	★★★	8 stars
Lui et al. 2014 [10]	★★★★	★	★★ c)	7 stars
Song et al. 2018 [11]	★★★★	★	★★★	8 stars
Kim D et al. 2020 [54]	★★★★	★	★★★	8 stars
Huang et al. 2020 [52]	★★★ a)	★	★★ c)	6 stars
Yoo et al. 2020 [17]	★★★ b)	★	★★ c)	6 stars

a) The exposed cohort was not representative because there were only 4 roots with periodontal involvement in a total sample of 95 roots; b) The exposed cohort was not representative because there were only 9 teeth with periodontal involvement in a total sample of 225 teeth; c) Recall rate < 50%.
