# Supplementary material for: Impact of Periodontal Attachment Loss on the Outcome of Endodontic Microsurgery: A Systematic Review and Meta-Analysis
Source: Medicina (Kaunas). 2021 Sep 1;57(9):922. doi: 10.3390/medicina57090922 (PMC8465214; doi:10.3390/medicina57090922)
Supplement: Supplementary file 1 [file medicina-57-00922-s001.zip › Table S5.pdf]

Table S5. Risk of bias of cohort studies based on ROBINS-I tool, as proposed by Cochrane.

Study	D1 Bias due to confounding	D2 Bias in selecting participants for the study	D3 Bias in classifying interventions	D4 Bias due to deviations from intended intervention	D5 Bias due to missing data	D6 Bias in measuring outcomes	D7 Bias in selecting reported result	Overall judgement
von Arx et al. 2007 [14]	LOW	LOW	LOW	LOW	LOW	MODERATE [c)]	LOW	LOW
Kim E et al. 2008 [15]	LOW	LOW	LOW	LOW	LOW	LOW	LOW	LOW
Song et al. 2013 [51]	LOW	LOW	LOW	LOW	MODERATE [a)]	MODERATE [c)]	LOW	MODERATE
Song et al. 2013 [13]	LOW	LOW	LOW	LOW	LOW	MODERATE [c)]	LOW	LOW
Song et al. 2011 [26]	LOW	LOW	LOW	LOW	MODERATE [a)]	MODERATE [c)]	LOW	MODERATE
von Arx et al. 2012 [53]	LOW	LOW	LOW	LOW	LOW	LOW	LOW	LOW
Song et al. 2012 [9]	LOW	LOW	LOW	LOW	MODERATE [a)]	LOW	LOW	LOW
Lui et al. 2014 [10]	LOW	LOW	LOW	LOW	HIGH [b)]	LOW	LOW	HIGH
Song et al. 2018 [11]	LOW	LOW	LOW	LOW	LOW	MODERATE [c)]	LOW	LOW
Kim D et al. 2020 [54]	LOW	LOW	LOW	LOW	LOW	MODERATE [c)]	LOW	LOW
Huang et al. 2020 [52]	LOW	LOW	LOW	LOW	HIGH [b)]	LOW	LOW	HIGH
Yoo et al. 2020 [17]	LOW	LOW	LOW	LOW	HIGH [b)]	MODERATE [c)]	LOW	HIGH

[a)] Recall rate between 50% and 70%; [b)] Recall rate < 50%; [c)] The outcome assessors were not blind to the intervention received by study's participants.
